# *cis*-Bis(1,10-phenanthroline-κ^2^ *N*,*N*′)bis­(thio­cyanato-κ*N*)magnesium(II)

**DOI:** 10.1107/S1600536810027054

**Published:** 2010-07-21

**Authors:** Dan Zhao, Fei-Fei Li, Jiao Sha

**Affiliations:** a Department of Physics and Chemistry, Henan Polytechnic University, Jiaozuo, Henan 454000, People’s Republic of China

## Abstract

The title compound, [Mg(NCS)_2_(C_12_H_8_N_2_)_2_], has been synthesized from the hydro­thermal reaction of MgCl_2_, KSCN, 1,10-phenanthroline and H_2_O. Its structure is isotypic with the Mn^II^, Fe^II^, Co^II^, Ni^II^, Cu^II^ and Zn^II^ analogues. The Mg^II^ cation has a slightly distorted octa­hedral geometry containing four N atoms from two 1,10-phenanthroline mol­ecules and two N atoms from two thio­cyanate anions. The asymmetric unit contains one-half mol­ecule, and the complete complex has 2 symmetry.

## Related literature

For isotypic compounds with transition metals, see: Baker & Bobonich (1964 ▶); Gallois *et al.* (1990 ▶); Ganguli *et al.* (1981 ▶); Gütlich (1981 ▶); König (1968 ▶); Holleman *et al.* (1994 ▶); Yin (2007 ▶); Freire *et al.* (2001 ▶); Kabešová & Kožíšková (1992 ▶); Parker *et al.* (1996 ▶); Liu *et al.* (2005 ▶). 
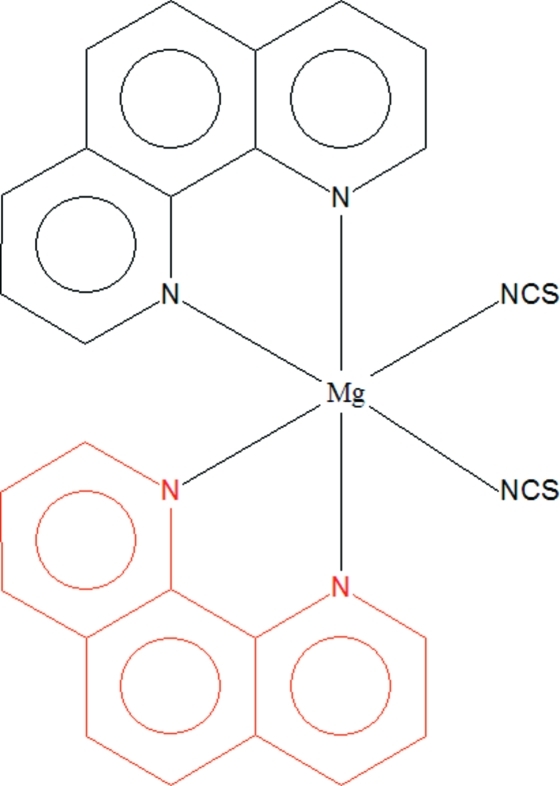


## Experimental

### 

#### Crystal data

[Mg(NCS)_2_(C_12_H_8_N_2_)_2_]
*M* *_r_* = 500.88
Orthorhombic, 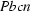

*a* = 13.2159 (3) Å
*b* = 10.1426 (2) Å
*c* = 17.4783 (3) Å
*V* = 2342.85 (8) Å^3^
*Z* = 4
Mo *K*α radiation
μ = 0.28 mm^−1^
*T* = 296 K
0.30 × 0.25 × 0.25 mm

#### Data collection

Bruker APEXII CCD diffractometer
Absorption correction: multi-scan (*SADABS*; Bruker, 2007 ▶) *T*_min_ = 0.920, *T*_max_ = 0.933
10066 measured reflections
2168 independent reflections
1631 reflections with *I* > 2σ(*I*)
*R*_int_ = 0.024

#### Refinement

*R*[*F* ^2^ > 2σ(*F* ^2^)] = 0.036
*wR*(*F* ^2^) = 0.098
*S* = 1.03
2168 reflections
159 parameters
1 restraint
H-atom parameters constrained
Δρ_max_ = 0.16 e Å^−3^
Δρ_min_ = −0.23 e Å^−3^

### 

Data collection: *APEX2* (Bruker, 2007 ▶); cell refinement: *SAINT-Plus* (Bruker, 2007 ▶); data reduction: *SAINT-Plus*; program(s) used to solve structure: *SHELXS97* (Sheldrick, 2008 ▶); program(s) used to refine structure: *SHELXL97* (Sheldrick, 2008 ▶); molecular graphics: *SHELXTL* (Sheldrick, 2008 ▶); software used to prepare material for publication: *SHELXTL*.

## Supplementary Material

Crystal structure: contains datablocks I, global. DOI: 10.1107/S1600536810027054/bh2298sup1.cif

Structure factors: contains datablocks I. DOI: 10.1107/S1600536810027054/bh2298Isup2.hkl

Additional supplementary materials:  crystallographic information; 3D view; checkCIF report

## Figures and Tables

**Fig. 1.**
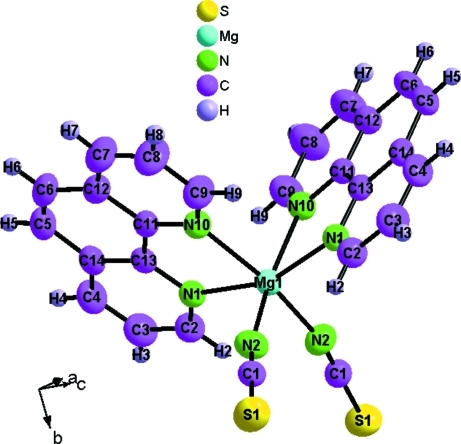
The molecular structure of the title compound. Displacement ellipsoids are drawn at the 50% probability level for non-H atoms.

## supplementary crystallographic information

### Comment

Metallorganic compounds with the general formula [*M*(NCS)_2_(C_12_H_8_N_2_)_2_] (*M*=Mn^II^, Fe^II^, Co^II^, Ni^II^, Cu^II^ and Zn^II^) have been studied for many decades. Thereinto, [Fe(NCS)_2_(C_12_H_8_N_2_)_2_] is reported to be one of the prototypical spin crossover compounds and its magnetic properties have been most investigated by various techniques (Baker & Bobonich, 1964; Gallois *et al.*, 1990; Ganguli *et al.*, 1981; Gütlich, 1981; König, 1968). Henceforth, isostructural compounds for Mn^II^ (Holleman *et al.*, 1994), Co^II^ (Yin, 2007), Ni^II^ (Freire *et al.*, 2001), Cu^II^ (Kabešová & Kožíšková, 1992; Parker *et al.*, 1996) and Zn^II^ (Liu *et al.*, 2005) analogues have been prepared and their structures have been studied. However, as far as our knowledge goes, the crystal structure for Mg^II^ analogue has not been reported so far. Herein, we report the single-crystal structure of the magnesium complex [Mg(NCS)_2_(C_12_H_8_N_2_)_2_] prepared by hydrothermal reaction.

The molecular structure of the title compound is shown in Fig. 1. The coordination geometry of the Mg^II^ ion is distorted octahedral, in which four positions are occupied by four N atoms of two chelating phen ligands and the other two occupied by two N atoms of two thiocyanate ligands with a *cis* arrangement. The Mg—N_phen_ and Mg—N_thiocyanate_ bond lengths are 2.2151 (15), 2.2253 (16) and 2.0844 (18) Å, respectively.

### Experimental

A mixture of MgCl_2_ (0.05 g), KSCN (0.1 g), 1,10-phenanthroline (0.1 g) and H_2_O (15 ml), was sealed in a 25 ml Teflonlined bomb at 448 K for 7 days and then cooled to room temperature. Colorless prismatic crystals were obtained in low yield.

### Refinement

Constraint instruction 'DELU 0.005 C1 S1' was used in the refinement. The final difference map shows that the highest peak is 0.16 e/Å^3^ at 1.00 Å from S1, while the deepest hole is -0.26 e/Å^3^ at 0.55 Å from S1, too. All H atoms were placed in idealized positions with C—H bond lengths constrained to 0.93 Å and *U*_iso_(H)=1.2*U*_eq_(carrier C atom).

### Crystal data

**Table:**

[Mg(NCS)_2_(C_12_H_8_N_2_)_2_]	*F*(000) = 1032
*M**_r_* = 500.88	*D*_x_ = 1.420 Mg m^−^^3^
Orthorhombic, *P**b**c**n*	Mo *K*α radiation, λ = 0.71073 Å
Hall symbol: -P 2n 2ab	Cell parameters from 2890 reflections
*a* = 13.2159 (3) Å	θ = 2.5–24.9°
*b* = 10.1426 (2) Å	µ = 0.28 mm^−^^1^
*c* = 17.4783 (3) Å	*T* = 296 K
*V* = 2342.85 (8) Å^3^	Prism, colourless
*Z* = 4	0.30 × 0.25 × 0.25 mm

### Data collection

**Table:**

Bruker APEXII CCD diffractometer	2168 independent reflections
Radiation source: fine-focus sealed tube	1631 reflections with *I* > 2σ(*I*)
graphite	*R*_int_ = 0.024
φ and ω scans	θ_max_ = 25.4°, θ_min_ = 2.3°
Absorption correction: multi-scan (*SADABS*; Bruker, 2007)	*h* = −15→12
*T*_min_ = 0.920, *T*_max_ = 0.933	*k* = −12→7
10066 measured reflections	*l* = −18→21

### Refinement

**Table:**

Refinement on *F*^2^	Primary atom site location: structure-invariant direct methods
Least-squares matrix: full	Secondary atom site location: difference Fourier map
*R*[*F*^2^ > 2σ(*F*^2^)] = 0.036	Hydrogen site location: inferred from neighbouring sites
*wR*(*F*^2^) = 0.098	H-atom parameters constrained
*S* = 1.03	*w* = 1/[σ^2^(*F*_o_^2^) + (0.0449*P*)^2^ + 0.7099*P*] where *P* = (*F*_o_^2^ + 2*F*_c_^2^)/3
2168 reflections	(Δ/σ)_max_ < 0.001
159 parameters	Δρ_max_ = 0.16 e Å^−^^3^
1 restraint	Δρ_min_ = −0.23 e Å^−^^3^
0 constraints	

### Special details

**Table:**

Refinement. Constraint instruction 'DELU 0.005 C1 S1' was used in the refinement to minimize the large differences in the anisotropic displacement parameters along the C—S bond in the thiocyanate group.

### Fractional atomic coordinates and isotropic or equivalent isotropic displacement parameters (Å^2^)

**Table:**

	*x*	*y*	*z*	*U*_iso_*/*U*_eq_	
S1	0.64249 (5)	0.47752 (6)	0.07238 (3)	0.0661 (2)	
Mg1	0.5000	0.16430 (8)	0.2500	0.0383 (2)	
N1	0.66107 (11)	0.12610 (14)	0.27718 (9)	0.0407 (4)	
C1	0.58353 (15)	0.37621 (18)	0.12830 (11)	0.0433 (5)	
N2	0.54082 (13)	0.30322 (16)	0.16772 (10)	0.0530 (4)	
C2	0.74157 (15)	0.18248 (18)	0.24607 (11)	0.0481 (5)	
H2	0.7314	0.2488	0.2102	0.058*	
C3	0.84039 (16)	0.1479 (2)	0.26420 (13)	0.0577 (6)	
H3	0.8945	0.1904	0.2407	0.069*	
C4	0.85730 (16)	0.0515 (2)	0.31650 (13)	0.0566 (6)	
H4	0.9231	0.0276	0.3293	0.068*	
C5	0.78512 (18)	−0.1150 (2)	0.40632 (12)	0.0571 (6)	
H5	0.8494	−0.1435	0.4202	0.069*	
C6	0.70375 (19)	−0.1715 (2)	0.43840 (12)	0.0597 (6)	
H6	0.7128	−0.2379	0.4744	0.072*	
C7	0.51610 (19)	−0.1879 (2)	0.44975 (14)	0.0689 (7)	
H7	0.5213	−0.2551	0.4857	0.083*	
C8	0.4239 (2)	−0.1440 (2)	0.42750 (15)	0.0723 (7)	
H8	0.3654	−0.1794	0.4489	0.087*	
C9	0.41727 (17)	−0.0456 (2)	0.37250 (13)	0.0573 (6)	
H9	0.3534	−0.0165	0.3579	0.069*	
N10	0.49728 (12)	0.00873 (15)	0.33976 (9)	0.0441 (4)	
C11	0.59014 (15)	−0.03253 (17)	0.36322 (10)	0.0409 (4)	
C12	0.60330 (17)	−0.13185 (19)	0.41840 (11)	0.0502 (5)	
C13	0.67710 (14)	0.02912 (17)	0.32956 (10)	0.0391 (4)	
C14	0.77482 (15)	−0.01174 (18)	0.35110 (11)	0.0464 (5)	

### Atomic displacement parameters (Å^2^)

**Table:**

	*U*^11^	*U*^22^	*U*^33^	*U*^12^	*U*^13^	*U*^23^
S1	0.0746 (5)	0.0616 (4)	0.0620 (4)	−0.0142 (3)	0.0094 (3)	0.0093 (3)
Mg1	0.0326 (5)	0.0381 (4)	0.0441 (5)	0.000	−0.0008 (4)	0.000
N1	0.0369 (9)	0.0411 (8)	0.0442 (8)	0.0004 (7)	−0.0021 (7)	−0.0023 (7)
C1	0.0423 (11)	0.0413 (10)	0.0464 (11)	0.0071 (9)	−0.0031 (9)	−0.0029 (9)
N2	0.0500 (11)	0.0498 (9)	0.0592 (11)	0.0024 (8)	0.0044 (9)	0.0091 (9)
C2	0.0391 (11)	0.0512 (11)	0.0542 (12)	−0.0026 (9)	0.0011 (9)	0.0013 (10)
C3	0.0371 (11)	0.0646 (13)	0.0714 (15)	−0.0005 (10)	0.0035 (10)	−0.0037 (12)
C4	0.0344 (12)	0.0650 (13)	0.0705 (15)	0.0097 (10)	−0.0071 (10)	−0.0075 (12)
C5	0.0543 (14)	0.0574 (13)	0.0597 (13)	0.0124 (11)	−0.0188 (11)	−0.0034 (11)
C6	0.0707 (16)	0.0539 (12)	0.0545 (13)	0.0126 (12)	−0.0174 (12)	0.0082 (11)
C7	0.0724 (17)	0.0671 (15)	0.0671 (15)	−0.0040 (13)	−0.0048 (13)	0.0271 (12)
C8	0.0616 (16)	0.0769 (16)	0.0785 (16)	−0.0115 (13)	0.0059 (13)	0.0314 (14)
C9	0.0442 (12)	0.0620 (13)	0.0656 (14)	−0.0032 (10)	0.0021 (11)	0.0149 (11)
N10	0.0390 (9)	0.0446 (8)	0.0486 (9)	−0.0017 (7)	−0.0009 (7)	0.0036 (7)
C11	0.0469 (11)	0.0377 (9)	0.0381 (10)	0.0042 (8)	−0.0067 (9)	−0.0038 (8)
C12	0.0603 (14)	0.0454 (11)	0.0450 (11)	0.0019 (10)	−0.0071 (10)	0.0030 (9)
C13	0.0402 (11)	0.0382 (9)	0.0390 (10)	0.0027 (8)	−0.0047 (8)	−0.0080 (8)
C14	0.0446 (12)	0.0469 (11)	0.0478 (11)	0.0086 (9)	−0.0104 (9)	−0.0107 (9)

### Geometric parameters (Å, °)

**Table:**

S1—C1	1.618 (2)	C5—C6	1.341 (3)
Mg1—N2	2.0843 (18)	C5—C14	1.431 (3)
Mg1—N2^i^	2.0844 (18)	C5—H5	0.9300
Mg1—N1	2.2151 (15)	C6—C12	1.431 (3)
Mg1—N1^i^	2.2152 (15)	C6—H6	0.9300
Mg1—N10^i^	2.2253 (16)	C7—C8	1.355 (3)
Mg1—N10	2.2254 (16)	C7—C12	1.397 (3)
N1—C2	1.325 (2)	C7—H7	0.9300
N1—C13	1.360 (2)	C8—C9	1.388 (3)
C1—N2	1.158 (2)	C8—H8	0.9300
C2—C3	1.389 (3)	C9—N10	1.323 (3)
C2—H2	0.9300	C9—H9	0.9300
C3—C4	1.357 (3)	N10—C11	1.360 (2)
C3—H3	0.9300	C11—C12	1.405 (3)
C4—C14	1.402 (3)	C11—C13	1.434 (3)
C4—H4	0.9300	C13—C14	1.408 (3)
			
N2—Mg1—N2^i^	94.93 (10)	C6—C5—H5	119.4
N2—Mg1—N1	91.00 (6)	C14—C5—H5	119.4
N2^i^—Mg1—N1	102.65 (6)	C5—C6—C12	121.44 (19)
N2—Mg1—N1^i^	102.65 (6)	C5—C6—H6	119.3
N2^i^—Mg1—N1^i^	91.00 (6)	C12—C6—H6	119.3
N1—Mg1—N1^i^	159.85 (9)	C8—C7—C12	119.7 (2)
N2—Mg1—N10^i^	89.35 (6)	C8—C7—H7	120.1
N2^i^—Mg1—N10^i^	165.91 (6)	C12—C7—H7	120.1
N1—Mg1—N10^i^	90.67 (6)	C7—C8—C9	119.4 (2)
N1^i^—Mg1—N10^i^	74.95 (6)	C7—C8—H8	120.3
N2—Mg1—N10	165.91 (6)	C9—C8—H8	120.3
N2^i^—Mg1—N10	89.35 (6)	N10—C9—C8	123.3 (2)
N1—Mg1—N10	74.95 (6)	N10—C9—H9	118.3
N1^i^—Mg1—N10	90.67 (6)	C8—C9—H9	118.3
N10^i^—Mg1—N10	89.69 (9)	C9—N10—C11	117.57 (16)
C2—N1—C13	117.60 (16)	C9—N10—Mg1	127.84 (14)
C2—N1—Mg1	127.47 (13)	C11—N10—Mg1	114.59 (12)
C13—N1—Mg1	114.86 (12)	N10—C11—C12	122.62 (19)
N2—C1—S1	179.34 (19)	N10—C11—C13	117.73 (16)
C1—N2—Mg1	165.63 (16)	C12—C11—C13	119.65 (18)
N1—C2—C3	123.54 (19)	C7—C12—C11	117.3 (2)
N1—C2—H2	118.2	C7—C12—C6	123.72 (19)
C3—C2—H2	118.2	C11—C12—C6	119.0 (2)
C4—C3—C2	119.4 (2)	N1—C13—C14	122.40 (18)
C4—C3—H3	120.3	N1—C13—C11	117.80 (17)
C2—C3—H3	120.3	C14—C13—C11	119.80 (17)
C3—C4—C14	119.49 (19)	C4—C14—C13	117.60 (18)
C3—C4—H4	120.3	C4—C14—C5	123.50 (19)
C14—C4—H4	120.3	C13—C14—C5	118.90 (19)
C6—C5—C14	121.2 (2)		

Symmetry codes: (i) −*x*+1, *y*, −*z*+1/2.
